# OA22 A case report of Whipple's arthritis

**DOI:** 10.1093/rap/rkac066.022

**Published:** 2022-09-28

**Authors:** Khizer Iqbal, Zahra Haider, Gayle Smithson

**Affiliations:** The Midyorkshire NHS Trust, Wakefield, United Kingdom; The Midyorkshire NHS Trust, Wakefield, United Kingdom; The Midyorkshire NHS trust, Wakefield, United Kingdom

## Abstract

**Introduction/Background:**

Whipple’s disease is rare chronic systemic infection caused by the Tropheryma whipplei. Initially it was thought it can only affect the Gastrointestinal tract but later on it was discovered that it is multisystemic and can affect heart, brain, skin, eyes and lungs. The common presenting features are gastrointestinal tract symptoms like diarrhoea, vomiting, reduced appetite and weight loss. Other common extra intestinal features are joints pain, uveitis, and confusion and memory loss. Rarely, it can present with seizures and other neurological deficits. It predominantly affects the males as compared to females likely with defective T-Lymphocytes particularly TH1 population function.

**Description/Method:**

A 69 year old gentleman presented with unexplained weight loss, chronic diarrhoea, reduced appetite and abdominal bloating with travelling history in the past. Background history of epilepsy, hypercholesterolemia and osteoarthritis. Initial blood tests showed a normocytic anaemia along with lymphopenia. CT thorax and abdomen found indeterminate mesenteric lymphadenopathy. Upper GI endoscopy confirmed tiny proximal stomach tiny polyps, polypectomy was done along with multiple biopsies taken from duodenum. The duodenal biopsies histopathology report had shown an infiltration of PAS stain positive foamy macrophages with mixed inflammatory cells within the lamina propria along with broadening of villi. Furthermore, no micro-organisms were seen, including fungi and mycobacteria along with atypical cells. Histopathology is quite consistent with Whipple's disease however tissue biopsy for Whipple’s PCR was negative but serum Whipple’s PCR was detected. On infectious disease team advice he was started on ceftriaxone for two weeks later, switched to oral trimoxazole for 12 months resulted in complete symptoms resolution along with negative Whipple's PCR.

Three months later, Rheumatology team was involved as he had developed palindromic migratory polyarthritis along with raised inflammatory markers, involving his right knee, right wrist, left elbow and then left wrist along with active joint inflammation. Blood investigations had shown weakly positive Rheumatoid factor with negative anti CCP and ANA. The plain films of various joints did not show any early inflammatory changes like periarticular osteopenia and erosions. Infectious disease team had suggested a repeat endoscopy reassuringly, he had negative serum and biopsies Whipple's PCR. After couple of weeks he developed a left knee pain with joint effusion which was aspirated with aseptic technique and the left knee synovial fluid came positive for Whipple’s PCR. On Infectious team recommendation he was commenced on hydroxychloroquine and doxycycline for another 12 months.

**Discussion/Results:**

Whipple’s disease arthritis is easily misdiagnosed and approximately 1.58% of patients with unexplained seronegative inflammatory arthritis are positive for Tropheryma whipplei. Furthermore, the diagnosis of Whipple’s disease can be prolonged in patients with no gastrointestinal tract symptoms and in some cases gastrointestinal symptoms occur after extra intestinal features. In 41%to 61% cases of Whipple’s disease arthritis was reported which indicates Whipple's disease is significantly associated with arthritis and even more in Whipple's prodromal disease where the percentages of joint symptoms peak around 40%-80%. With the antibiotics the outcome of Whipple's disease can be fatal, it can be the worst when patients are misdiagnosed and on immunosuppressants.

Giving a reference of retrospective single centre cohort study of seven patients was done. All 7 patients presented with symmetrical polyarthritis and 3 patients developed erosive arthritis. The most common joints were wrists, knuckles and knees involved, less commonly joints involvement were hips, elbows and shoulders. All patients were seronegative and out of 7, 6 patients were given disease modifying agents for considering seronegative rheumatoid arthritis but didn’t get good treatment response. All patients were treated with treated with ceftriaxone along with Co –Trimoxazole or Doxycycline/hydroxychloroquine. Both combinations were used as alternatives due to side effects or inefficacy and eventually lead to arthritis remission over the treatment course of 12 - 27 months. Interestingly, 2 out of 6 patients duodenal biopsies were positive for PAS staining and but all patients synovial fluid from one inflamed joint was positive for Whipple's PCR.

The desirable investigations for suspected Whipple's arthritis are for suspected Whipple’s disease are PCR with samples of joint fluid, saliva and stools.

In our case, Patient joints symptoms resolved with the course of antibiotics without progression of further joint involvement.

**Key learning points/Conclusion:**

In conclusion, Whipple’s disease can rarely present like seronegative arthritis with or without GI symptoms. For patients with seronegative inflammatory arthritis not responding to initial DMARDs therapy, Whipple’s arthritis should be excluded. The positive outcome about Whipple’s arthritis is that it is antimicrobial treatment responsive. In the suspected patients diagnostics test to rule out Whipple's arthritis is minimal invasive synovial fluid tap for Whipple's PCR, which can be performed in routine clinical practice.

